# Brain networks underlying aesthetic appreciation as modulated by interaction of the spectral and temporal organisations of music

**DOI:** 10.1038/s41598-019-55781-9

**Published:** 2019-12-19

**Authors:** Seung-Goo Kim, Karsten Mueller, Jöran Lepsien, Toralf Mildner, Thomas Hans Fritz

**Affiliations:** 1 Max Planck Institute for Human Cognitive and Brain Sciences, Leipzig, Germany; 2 Department of Psychology and Neuroscience, Duke University, Durham, NC United States; 3 Institute for Psychoacoustics and Electronic Music, University of Ghent, Ghent, Belgium

**Keywords:** Perception, Prefrontal cortex

## Abstract

Music is organised both spectrally and temporally, determining musical structures such as musical scale, harmony, and sequential rules in chord progressions. A number of human neuroimaging studies investigated neural processes associated with emotional responses to music investigating the influence of musical valence (pleasantness/unpleasantness) comparing the response to music and unpleasantly manipulated counterparts where harmony and sequential rules were varied. Interactions between the previously applied alterations to harmony and sequential rules of the music in terms of emotional experience and corresponding neural activities have not been systematically studied although such interactions are at the core of how music affects the listener. The current study investigates the interaction between such alterations in harmony and sequential rules by using data sets from two functional magnetic resonance imaging (fMRI) experiments. While replicating the previous findings, we found a significant interaction between the spectral and temporal alterations in the fronto-limbic system, including the ventromedial prefrontal cortex (vmPFC), nucleus accumbens, caudate nucleus, and putamen. We further revealed that the functional connectivity between the vmPFC and the right inferior frontal gyrus (IFG) was reduced when listening to excerpts with alterations in both domains compared to the original music. As it has been suggested that the vmPFC operates as a pivotal point that mediates between the limbic system and the frontal cortex in reward-related processing, we propose that this fronto-limbic interaction might be related to the involvement of cognitive processes in the emotional appreciation of music.

## Introduction

Music has been ubiquitous in human cultures for more than 40,000 years^1^, presumably, at least partly, for its hedonic value^2–4^. As a form of art that uses sound as a medium, music embodies unique spectral structures (e.g. musical scale systems, tonal hierarchy, timbres of musical instruments) and temporal structures (e.g. sequential rules in the progression of chords, voice leading, motif development, dynamic rhythms, regular tempo). In the discourse on aesthetical experience of music from the perspective of cognitive neuroscience, the affective influence of harmony (either consonant or dissonant) was suggested to render sensory pleasantness, or “core liking^5,6^”, whereas temporal structures of music, or sequential rules, were proposed to rather be evaluated via “cognitive processing”, which extracts rules from a sequence of sound and creates expectancy.

Dissonant harmony was associated with decreased functional signal (e.g. regional cerebral blood flow [rCBF] or blood oxygen level dependent [BOLD] signal) in the temporal and frontal cortices including the bilateral superior temporal gyrus (STG), frontal opercula, and insulae^7–11^. A violation of expectancy based on a sequential rule evoked synchronised neural responses in the inferior frontal gyrus (IFG) mostly in the right hemisphere^12–16^. Furthermore, scrambling the temporal order of musical excerpts resulted in decreased BOLD signal in the ventral striatum and the orbitofrontal cortex^17^, which seems to support the notion that a major underlying mechanism of music-induced pleasure is based on tension that is built up by violation of expectation and its prolonged fulfilment^18,19^.

While a number of previous studies used disruption in either a spectral or temporal structure to create unpleasant counterparts to investigate music-induced emotions, the interaction between these musical structures remains largely unknown. If aesthetical appreciation of music were indeed a holistic process as has been suggested^6^, one would expect that the processing of both structures is closely related and that disrupting the processing of one would disrupt the processing of the other. In other words, a disruption of spectral structure for example would alter neural responses differently depending on the temporal structure. As suggested in previous publications^3,20^, it is probable that these spectral and temporal structures of music are integrated in association cortices (i.e. ventromedial prefrontal cortex [vmPFC] or IFG) corresponding to a type of “higher-order” emotional processing distinct from a more “lower-order” auditory signal processing along the auditory pathway (e.g. inferior colliculus or STG). To test this conjecture, we investigated an interaction between spectral and temporal structures in music using functional magnetic resonance imaging (fMRI) data from two human experiments that are different in MR sequences, mutually complementarily capturing brain activities related to music perception. We were especially interested in areas that showed interaction effects in integrating spectral and temporal dimensions in music.

## Materials and Methods

### Stimuli

Musical excerpts were extracted from instrumental music from the last four centuries, which have been or had been popular to general audience. Musical styles included classical (e.g. J. S. Bach), swing (e.g. Benny Goodman), and tango (e.g. Francisco Canaro) as used in previous studies^11,21^ (see Supplementary Table S1 for a full list of excerpts). The musical excerpts were manipulated in the spectral structure (i.e. harmony) and the temporal structure (i.e. play direction) resulting in an orthogonal 2 × 2 factorial design. To alter the spectral structure, the original excerpt was transposed two semitones up (i.e. major second) and six semitones down (i.e. diminished fifth), and subsequently mixed together, resulting in added dissonant intervals throughout the excerpts that affect local harmony and tonal context. To alter the temporal structure, the excerpt was played backward. This resulted in changes in musical timbre, locally, and direction of chord progressions, more globally. All stimuli across conditions were controlled for loudness by equalizing the root-mean-square of the waveforms.

It is important to note that these physical manipulations were orthogonal in the sense that the change of spectral content (or temporal order) does not alter temporal order (or spectral content), but the results of manipulations are not musically independent. For example, the sequential rule of chord progression (e.g. frequent use of a tonic [the first chord of a diatonic scale] chord after a dominant [the fifth chord of a diatonic scale] at the end of a musical phrase) is based on tonal context (because it defines a tonic function of a triad [i.e. a major triad can be either a tonic, subdominant, or dominant of a major key]) as well as local harmony (because it forms simultaneous tones as a chord). Therefore, when the spectral manipulation makes the chords dissonant, expectation based on tonal context can be weakened, which would make the effect of reversal less salient.

**Figure 1:**
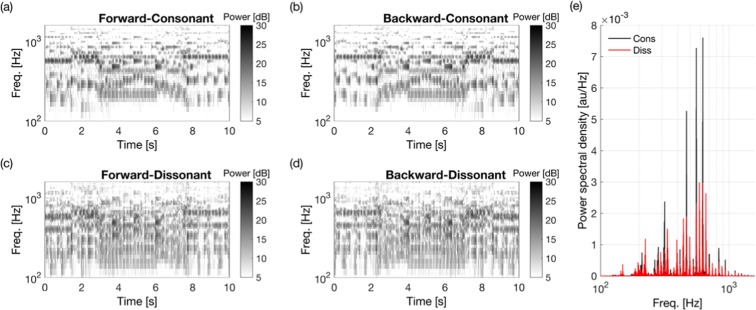
Spectrograms of four versions of a representative musical excerpt. Normalised power is visualised on the time-frequency plane for consonant (**a**,**b**) and dissonant (**c**,**d**) harmony; forward (**a**,**c**) and backward (**b**,**d**) temporal order. Power spectra of consonant and dissonant conditions are compared (**e**).

It is also noteworthy that these manipulations do not “completely” abolish musical organisation but proportionally degrade it. For illustration, spectrograms of four versions of a representative musical excerpt by J. S. Bach, played by Glenn Gould, are shown in Fig. 1 (download Supplementary File S1 for an audio file of the example). The conditions are labelled as “forward-consonant” (FC), “backward-consonant” (BC), “forward-dissonant” (FD), “backward-dissonant” (BD) for the play direction and the dissonance level. In general, the “dissonant” conditions (Fig. 1c,d) show more unresolved spectral components (thus perceived as dissonant) compared to “consonant” conditions (Fig. 1a,b). However, it is clear that the local spectral density in the dissonant excerpts fluctuates proportionally to the original harmony. Note that the condition labels (“consonant” and “dissonant”) are only relative descriptions; the original excerpts included both consonant and dissonant chords but the excerpts in “dissonant” conditions always had dissonant chords.

### Magnetic resonance imaging sequences

Functional neuroimaging data were adopted from two of our fMRI experiments, where the same stimuli but different MR sequences were used, thus providing us with complementary views of the brain activities (see Table 1 for an overview of the datasets). The first data set (“Experiment I”) has not been published elsewhere. The second data set (“Experiment II”) was used in two of our previous papers that revealed certain aspects of music perception that are different from the focus of the current study^7,10^; both studies addressed an investigation of the temporal dynamics of the ventral striatum, with respect to response to pleasant music^7^ and inter-subject correlation between the inferior colliculus response and subjective disliking of dissonant harmony^10^. The key difference between the two experiments was a silent delay between acquisition of fMRI volumes. In Experiment I, an fMRI volume was taken every 12 s (2 s to take one volume), allowing a silent period of 10 s without acoustic scanner noise, which was used to present auditory stimuli in the absence of acoustic noise from the MR sequence. This kind of MR sequence is known as “(temporally) sparse” scanning^22^. In Experiment II, fMRI volumes were “continuously” taken every second. Thus, in this experiment, subjects listened to musical stimuli in the presence of acoustic scanner noise.

**Table 1:** Participants’ Experiment parameters and characteristics from two experiments.

Metrics	Experiment I	Experiment II
Number of participants (female)	16 (14)	23 (13)
Age: mean ± std. [year]	25.8 ± 2.8	25.9 ± 2.9
Stimulus duration: mean ± std. (range) [s]	6.8 ± 2.0 (3.6–10)	30
TR (TA1) [s]	12 (2)	1 (1)
Number of volumes	233	2880
Duration of a trial: mean ± std. [s]	8.8 ± 2.0	36
Number of trials	200	80
Acquisition resolution [mm^3^]	3 × 3 × 5	2.5 × 2.5 × 4.5
Resampling resolution [mm^3^]	3 × 3 × 3	2.5 × 2.5 × 2.5
Smoothing kernel FWHM [mm^3^]	5 × 5 × 5	4 × 4 × 4
Average effective FWHM [mm^3^]	8.2 × 8.1 × 8.3	8.5 × 8.3 × 9.4
Volume of data space in the MNI152 space [liter]	1.324 (whole brain)	1.102 (ventral half)
Average number of resels	3284.1	1556.1

Abbreviation: std., standard deviation; TR, time of repetition; TA1, time of acquisition of one volume; FHWM, full width at half maximum, MNI, Montreal Neurological Institute.

Contamination of auditory stimuli by the acoustic noise of the MRI sequence is a serious problem in auditory fMRI experiments. In principle, given the delay of canonical hemodynamic function, acoustic contamination would be minimal with a non-scanning interval of 8 s, particularly when studying the primary auditory cortex^23^. However, this is an inefficient way to acquire fMRI data in terms of the number of volumes per given experiment time. Shortening the duration of a silent delay may increase statistical power by the increased number of samples (i.e. volumes), although it may also increase the interference by the acoustic scanner noise. A technical report study systematically compared sparse, alternating, and continuous sampling and reported non-linear alteration of auditory processing due to the presence of scanning noise^24^. Moreover, there is evidence that the effect of scanner noise is not limited to the primary auditory cortex but also to non-primary auditory cortices when processing spoken language^25^.

While the acoustic scanner noise is an issue that is not to be dealt with lightly, recent studies demonstrated that tonotopy experiments using continuous scanning can be successful with phase-encoded stimuli (i.e. frequency sweeping) and modern sound delivery systems^26,27^. Moreover, fMRI data at a higher temporal resolution enable us to investigate dynamic aspects of brain responses, particularly to dynamically evolving stimuli such as music. In our previous report of Experiment II^28^, we showed that continuous scanning yielded comparable results as the sparse scanning: a number of structures, including the cortical limbic areas and striatal regions, involved emotional appreciation. Thus, we aimed to use advantages of both MR sequences: (1) a precise localisation of involved brain regions without scanner noise using sparse sampling data (i.e. Experiment I) and (2) investigation of temporal dynamics and functional connectivity of the identified brain regions using continuous sampling data (i.e. Experiment II).

In Experiment I, twenty-four axial slices of echo planar imaging (EPI) that cover the whole brain were acquired with an in-plane resolution of 3 × 3 mm^2^, a thickness of 4 mm, and an inter-slice gap of 1 mm, resulting in a resolution of 3 × 3 × 5 mm^3^. Functional and T1-weighted images (1 × 1 × 1 mm^3^) were obtained using a 3-T Magnetom Tim Trio scanner (Siemens, Erlangen, Germany).

In Experiment II, fifteen axial slices of EPI that cover the ventral half of the brain were acquired with an in-plane resolution of 2.5 × 2.5 mm^2^, a thickness of 4 mm, and an inter-slice gap of 0.5 mm, resulting in a resolution of 2.5 × 2.5 × 4.5 mm^3^. EPI images were acquired using a 3-T MedSpec 30/100 scanner (Bruker, Ettlingen, Germany) and a birdcage head coil, and T1-weighted images at unit-mm isotropic resolution were acquired using a 3-T Magnetom Tim Trio scanner (Siemens, Erlangen, Germany).

### Experimental paradigms

**Figure 2:**
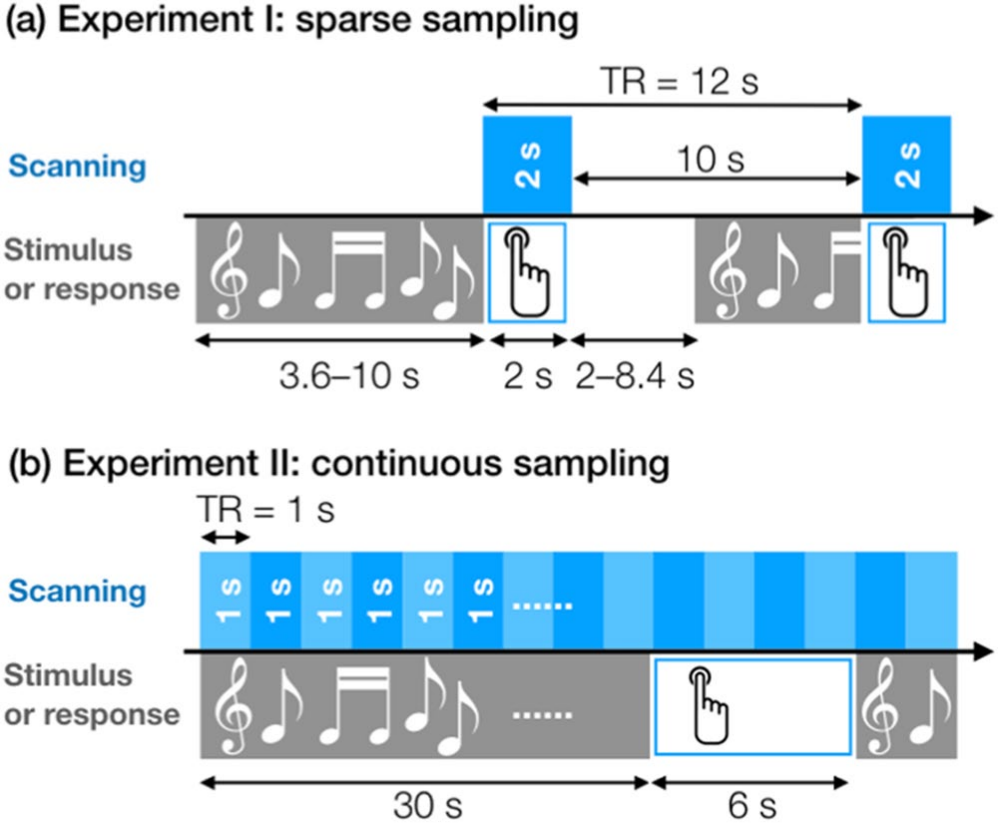
Schematic drawings depicting experimental paradigms of Experiment I (**a**) and Experiment II (**b**). Blue rectangles indicate acquisition of one fMRI volume. Grey shades with musical notations indicate presentation of musical stimuli. White rectangles with a pictogram of a hand pressing a button indicate the response time-window. Note that the duration of stimulus presentation and silent delay varied across trials in Experiment I (**a**).

Experiment paradigms of the two experiments are depicted in Fig. 2.

In Experiment I, one trial consisted of a silent period without scanning for 10 s and a scanning period for 2 s. The musical excerpts were presented during the silent period at pseudorandom timing (from 3.6 to 10 s with a step of 0.7 s before the acquisition of each volume) to sample different phases of the hemodynamic response to musical excerpts (namely, event-related design). Participants were instructed to press a button to rate subjective pleasantness of each excerpt (1 = very unpleasant, 2 = unpleasant, 3 = pleasant, 4 = very pleasant) during the 2-s scanning period. Twenty-five instrumental tunes were used to create 4 versions (FC, FD, BC, BD) and played twice, resulting in 25 × 4 × 2 = 200 trials.

In Experiment II, one trial consisted of a 30-s period for presentation of musical excerpts and a 6-s period for subjective rating of presented musical excerpts (36 s in total). Acoustic scanner noise was present throughout the whole experiment. Participants were instructed to press a button to rate subjective unpleasantness (1 = very pleasant, 2 = pleasant, 3 = unpleasant, 4 = very unpleasant) of each excerpt during the late 6-s period. Twenty instrumental tunes were played only once, resulting in 20 × 4 = 80 trials.

### Participants

Overall, 39 healthy volunteers participated in either one of two experiments (n = 16, 14 females, mean age 25.8 ± 2.8 years in Experiment I; n = 23, 13 females, mean age 25.9 ± 2.9 years in Experiment II). The studies were conducted strictly following guidelines approved by the Ethics Committee of the University of Leipzig. Informed written consent was obtained before the fMRI experiments. One participant in Experiment II was studying for a Bachelor’s degree in music, and other participants were students or professionals in non-musical fields. Some participants (four in Experiment I, sixteen in Experiment II) reported experience in playing musical instruments. However, the differences in the proportions of gender and musical experience were not statistically significant between the datasets (gender: *Z* = 0.29, *p* = 0.77; age: *T*(37) = −0.48, *p* = 0.64; musical experience: *Z* = 1.59, *p* = 0.11).

### Image processing

Using SPM12 (v6225; Wellcome Trust Centre for Neuroimaging, University Colleague of London, London, UK) and MATLAB (v8.6, R2015b; MathWorks, Natick, Massachusetts, USA), anatomical and functional images were processed, including unwarping and realignment, unified segmentation, spatial normalisation, and spatial smoothing. Because of the different TRs, slice-timing correction was done only for Experiment II. Also, for the same reason, we used 6 rigid body motion parameters and their lengths (i.e. L2-norm) of temporal derivatives of translation and rotation, respectively (i.e. 8 regressors in total) to regress out head movement artefacts in Experiment II. We resampled the functional data at isotropic resolutions that are close to the original resolutions^29^: 3-mm isotropic resolution for Experiment I; 2.5-mm for Experiment II. Different smoothing kernel sizes (full width at half maximum [FWHM] of 5 mm for Experiment I; 4 mm for Experiment II) were chosen to approximately match the effective smoothness of the two data sets at an isotropic FWHM of 8 mm. See Table 1 for detailed parameters.

### Functional activation analysis

A subject-level autoregressive general linear model (GLM) was carried out by encoding onsets and durations of four conditions (i.e. FC, FD, BC, and BD) for both data sets. Effects of conditions were estimated after adjustment for non-sphericity of the functional data using SPM12. Although the TR of Experiment I was very long (i.e. 12 s), because the images were obtained at various post-stimulus-onset times (from 3.6 to 10 s; namely event-related design), modelling hemodynamics in Experiment I is a valid approach. Because we previously reported that the ventral striatal response attenuated over time in Experiment II^7^, we modelled a 30-s condition into three 10-s segments and used the first segment to compute contrasts to match Experiment I. High-pass filter cut-off was 1/128 Hz for both experiments.

We computed multiple contrasts to test various effects: a partial effect of dissonance when excerpts were forward (FD - FC) or backward (BD - BC), a partial effect of reversal when excerpts were consonant (BC - FC) or dissonant (BD - FD), a joint effect of dissonance and reversal (BD - FC), and an interaction between the dissonance and reversal: (BD - BC) - (FD - FC).

A group-level one-sample *T*-test was carried out on subject-level contrast images. The Gaussian assumption was tested by carrying out a Kolmogorov–Sminov test at each voxel with false discovery rate correction. Because all corrected p-value in the brain mask was one, we used parametric inferences with random field theory (RFT)^30^ to control family-wise error rate (FWER) less than 0.05, as implemented in SPM12. The cluster-forming height-threshold was 0.001, and the extent-threshold was determined by the minimal extent of a cluster with a cluster-wise p-value less than 0.05, which was approximately 640 mm^3^ (24 voxels in Experiment I; 44 voxels in Experiment II). As pointed out by Flandin and Friston^31^, a recent criticism on cluster-extent thresholding by Eklund, *et al*.^32^ was based on results with liberal thresholds in height (uncorrected *p* = 0.01) and extent (80 mm^3^; only 3 voxels at 3-mm isotropic resolution). In the replication with a stringent height-threshold (uncorrected *p* = 0.001) by Flandin and Friston^31^, resulting FWERs were between 4 and 6%, demonstrating that the cluster-wise thresholding using SPM does not critically inflate FWER when employed carefully. Thus, we used conservative thresholds in both height and extent in the present study.

### Psychophysiological interaction analysis

In the functional activation analysis, the vmPFC showed similar BOLD activation to the most pleasant and the most unpleasant stimuli. Since the region showed sensitivity to disruptions in either spectral or temporal organisation of music in the current data, it is possible that what was altered was the functional connectivity of the vmPFC instead of the local activity. To test this idea, an analysis of psychophysiological interaction (PPI)^33^ was performed on the functional connectivity of the VMFPC using an SPM-based MATLAB toolbox for Generalized PPI^34^ (https://www.nitrc.org/projects/gppi). It has been known that a correlation between two BOLD time series is highly sensitive to abrupt and simultaneous changes in image intensities over many voxels, unlike BOLD activation analysis. Head motions during scanning may induce such signal changes, leading to spurious correlation^35^. Thus, for the PPI analysis, we employed 6 “anatomical CompCor” regressors, which are eigenvariates extracted from white matter and cerebrospinal fluid voxels to model non-neural global fluctuation in BOLD time series^36^. Also, for reliable estimation of functional connectivity, we used the whole 30-s trial for the PPI analysis. For a group-level statistical inference, the RFT was also used to control FWER to be less than 0.05.

## Results

### Experiment I

#### Partial effects of disrupted musical structures

**Figure 3:**
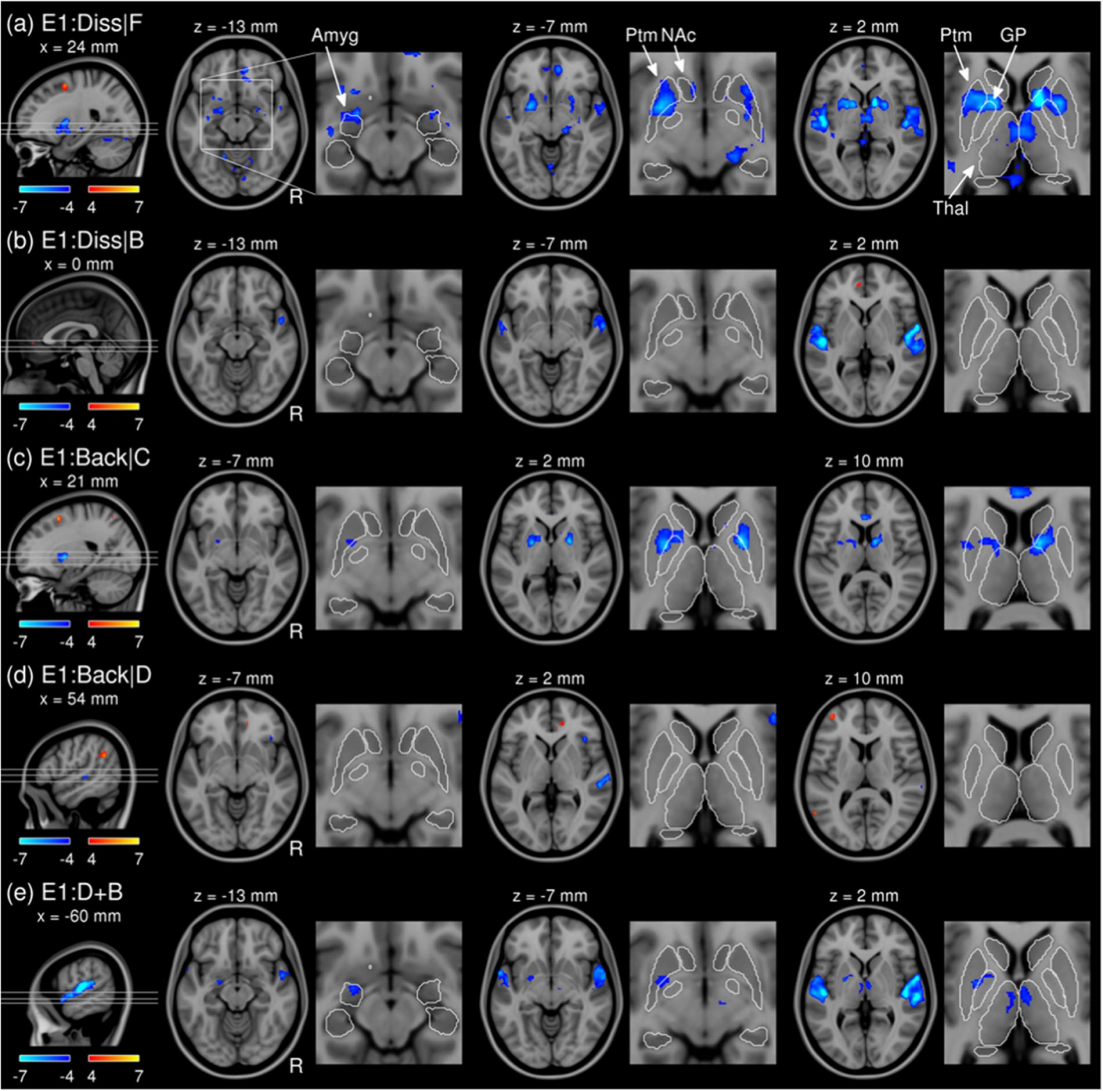
*T*-statistic maps (degrees of freedom of 15) of the partial effect of dissonance when forward (**a**) or backward (**b**), the partial effect of reversal when consonant (**c**) or dissonant (**d**), and the joint effect of disruption in both domains (**e**) in Experiment I. Zoomed views are given with contours of subcortical structures in white. Abbreviations: E1, Experiment I; Diss/D, dissonance; F, forward; Back/B, backward, C, consonant; Ptm, putamen; NAc, nucleus accumbens; GP, globus pallidus; CdN, caudate nucleus; Thal, thalamus.

We found decreases in the BOLD signal due to the dissonance when the excerpts were played forwards (i.e. FD – FC; Fig. 3a) in a number of brain regions, including the bilateral superior temporal gyri (STGs) and planum temporale (PT), the right planum polare (PP), the left amygdala and nucleus accumbens (NAc), the bilateral putamina (Ptm) and globus pallidi (GPs), and the medial parts of thalami (significant clusters are listed in Table 2). We also found a positive effect in the right superior frontal gyrus (SFG). Interestingly, the partial effect of dissonance when the excerpts were played backwards (i.e. BD - BC; Fig. 3b) was only significant in the auditory cortices (i.e. decreased BOLD signal in the bilateral STGs and the right PP), but not in the limbic areas (i.e. NAc, Ptm, or GPs).

**Table 2:** Significant clusters from Experiment I.

Structure name	Contrast value	Max *T* (15)	Max *Z*	Cluster *p*-value	Volume [cm^3^]	MNI-coord. [mm]		
						X	Y	Z
**(a) Partial effect of dissonance when forward**								
Right Superior Frontal Gyrus	0.283	5.554	4.033	0.038	0.6	24	2	56
Right Pallidum	−0.277	12.48	5.960	<10^−5^	16.7	12	5	2
Left Heschl’s Gyrus	−0.503	11.40	5.754	<10^−5^	13.0	−51	−10	5
Right Planum Temporale	−0.358	8.667	5.113	<10^−5^	10.8	57	−28	8
Left Cerebellum VI	−0.390	8.642	5.106	<10^−5^	7.7	−27	−61	−22
Right Cerebellum IX	−0.249	7.401	4.733	0.007	0.8	12	−55	−40
Left Cingulate Gyrus (anterior division)	−0.414	7.104	4.633	<10^−5^	4.5	−3	17	29
Right Thalamus	−0.307	6.038	4.236	0.002	1.0	15	−25	−4
Cerebellum Vermis VIIIa	−0.235	5.927	4.191	<10^−5^	1.6	3	−67	−37
Right Paracingulate Gyrus	−0.361	5.828	4.150	<10^−5^	2.3	9	44	−4
Right Lingual Gyrus	−0.713	5.539	4.026	<10^−5^	1.5	9	−82	−16
**(b) Partial effect of dissonance when backward**								
Left Paracingulate Gyrus	0.450	4.511	3.531	0.035	0.6	0	47	−1
Left Planum Temporale	−0.405	9.817	5.408	<10^−5^	8.5	−63	−28	8
Right Planum Polare	−0.430	9.373	5.299	<10^−5^	8.6	60	−1	2
Right Inferior Frontal Gyrus	−0.321	5.512	4.014	<10^−5^	2.2	39	14	23
**(c) Partial effect of reversal when consonant**								
Right Superior Frontal Gyrus	0.273	6.435	4.392	0.001	1.2	21	11	56
Right Lateral Occipital Cortex (superior division)	0.449	4.710	3.634	0.038	0.6	9	−64	62
Right Pallidum	−0.219	9.207	5.257	<10^−5^	2.8	18	2	5
Left Planum Temporale	−0.401	7.372	4.723	<10^−5^	1.3	−60	−34	17
Left Pallidum/Amygdala	−0.264	6.588	4.449	<10^−5^	2.7	−21	−1	2
Left Cingulate Gyrus (anterior division)	−0.456	6.164	4.287	<10^−5^	2.2	0	29	11
**(d) Partial effect of reversal when dissonant**								
Right Angular Gyrus	0.290	6.396	4.377	<10^−5^	1.30	54	−52	29
Left Angular Gyrus	0.460	6.118	4.269	<10^−5^	3.16	−54	−55	32
Right Cingulate Gyrus	0.336	6.065	4.247	0.037	0.59	12	41	−1
Left Middle Frontal Gyrus	0.224	5.915	4.186	0.024	0.65	−30	32	38
Left Frontal Pole	0.318	5.817	4.145	<10^−5^	1.62	−39	47	14
Right Superior Temporal Gyrus	−0.496	6.663	4.477	<10^−5^	1.40	57	−25	−1
Right Frontal Orbital Cortex	−0.222	6.508	4.420	0.024	0.65	36	26	−1
**(e) Joint effect of dissonance and reversal**								
Left Planum Temporale	−0.832	10.137	5.483	<10^−5^	12.7	−60	−22	11
Right Planum Polare	−0.580	9.611	5.358	<10^−5^	12.3	60	−1	2
Left Pallidum	−0.222	6.922	4.570	0.001	1.2	−21	−4	−1
Right Superior Frontal Gyrus	−0.237	6.196	4.299	0.003	1.0	9	−1	68
Right Thalamus	−0.282	5.239	3.891	0.001	1.1	6	−10	−1
**(f) Interaction between dissonance and reversal**								
Right Cingulate Gyrus (anterior division)	0.306	6.852	4.545	<10^−5^	7.8	3	23	20
Right Caudate/Nucleus Accumbens	0.384	6.603	4.455	<10^−5^	3.9	9	2	11
Left Angular Gyrus	0.156	6.491	4.413	0.018	0.7	−48	−58	38
Left Thalamus	0.256	5.964	4.206	<10^−5^	3.2	−12	−1	8
Right Cingulate Gyrus (posterior division)	0.226	5.663	4.080	<10^−5^	1.5	3	−28	41
Left Frontal Pole	0.228	5.060	3.806	0.002	1.0	−36	41	29
Right Thalamus	0.173	4.917	3.737	0.018	0.7	9	−22	5
Right Cingulate Gyrus (posterior division)	0.283	4.425	3.485	0.012	0.7	6	−46	35
Right Superior Frontal Gyrus	−0.269	5.242	3.892	0.012	0.7	24	2	53

Anatomical nomenclatures are based on the Harvard-Oxford atlases provided in FSL (https://fsl.fmrib.ox.ac.uk/).

The reversal of play direction when the excerpts were consonant (i.e. BC - FC; Fig. 3c) was associated with decreases in the BOLD signal in the bilateral Ptm, GP, and anterior cingulate cortex (ACC). We also found an increase in the right SFG similar to the partial effect of dissonance when played forwards. Similar to the analysis above, the partial effect of reversal when dissonant (i.e. BD - FD; Fig. 3d) was different from that when consonant. For the contrast BD – FD, we found decreases in the BOLD signal in the right PT and the right lateral orbital frontal cortex (OFC), but no change in the BOLD signal in the auditory cortices (i.e. STG, PT, and PP). We also found increases in the BOLD signal in a number of cortical regions, including the bilateral angular gyri, right ACC, left middle frontal gyrus (MFG), and the left frontal pole (FP). See Fig. S1 for all slices over the whole brain.

The joint effect of disruptions in spectral and temporal domains (i.e. BD - FC; Fig. 3e) was found as decreased BOLD signals in the bilateral STGs, the left GP, the anterior part of the left amygdala, and the medial parts of the bilateral thalami. Compared to the partial effects of either dissonance or reversal alone (Fig. 3a,c), the joint effect was weaker (i.e. less decrease in the BOLD signal) in the limbic system (i.e. NAc, Ptm, GP) and stronger (i.e., more decrease) in the auditory cortices.

Importantly, we found that the partial effect of one domain was dependent on the other domain. By definition, this implies an interaction between the two domains. We further quantitatively tested this observation in the following section.

#### Effect of interaction between disrupted musical structures in spectral vs. temporal domains

**Figure 4:**
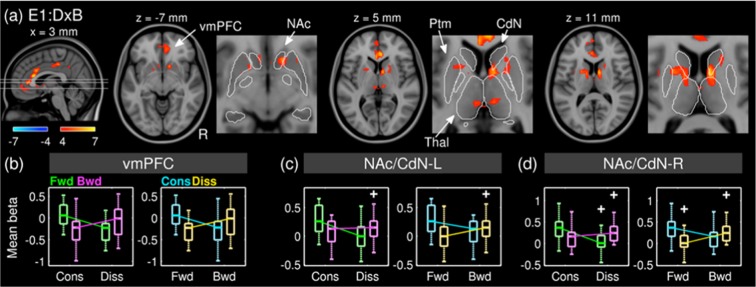
*T*-statistic maps (degrees of freedom of 15) for the interaction between dissonance and reversal (**a**) and boxplots showing effect sizes (i.e. beta coefficients) of four conditions averaged within clusters in the ventromedial prefrontal cortex (**b**), the left nucleus accumbens and caudate nucleus (**c**), and the right nucleus accumbens and caudate nucleus. (**d**) Abbreviations: E1, Experiment I; DxB, interaction between dissonance and backward; vmPFC, ventromedial prefrontal cortex; NAc, nucleus accumbens; Ptm, putamen; CdN, caudate nucleus; Thal, thalamus; Fwd, forward; Bwd, backward; Cons., consonant; Diss., dissonant.

We tested the interaction by subtracting the partial effect of dissonance when played backwards from that when played forwards; that is, (BD - BC) - (FD - FC). This is equivalent to the subtraction of the partial effect of reversal when dissonant from that when consonant because (BD - BC) - (FD - FC) = (BD - FD) - (BC - FC). We found a positive interaction in the ventromedial prefrontal cortex (vmPFC), ACC, and the subcortical limbic system including the NAc, the GPs, and thalami (Fig. 4a). This confirmed that the partial effect of a disruption in one domain was nullified when the other domain was disrupted in the cortical and subcortical limbic areas. In other words, a disruption in addition to a stimulus already with another disruption did not produce a further decrease in BOLD activation in the fronto-limbic areas. This was not the case in auditory regions.

To illustrate the GLM result in terms of effect size, beta coefficients averaged within each significant clusters are plotted in Fig. 4b–d. Interestingly, the positive interaction was so strong that the signs of marginal effects were flipped in the vmPFC and the bilateral striata. Indeed, the beta coefficients between the FC and BD conditions were not significantly different in all three clusters (max *T*(15) = 1.01; *p* > 0.10), which is surprising given the sensitivity to disrupted musical structures of the regions and the widely different acoustics and related emotional valances of conditions. We addressed this issue later in the analyses of PPI.

### Experiment II

#### Replication of the functional activation analysis

**Figure 5:**
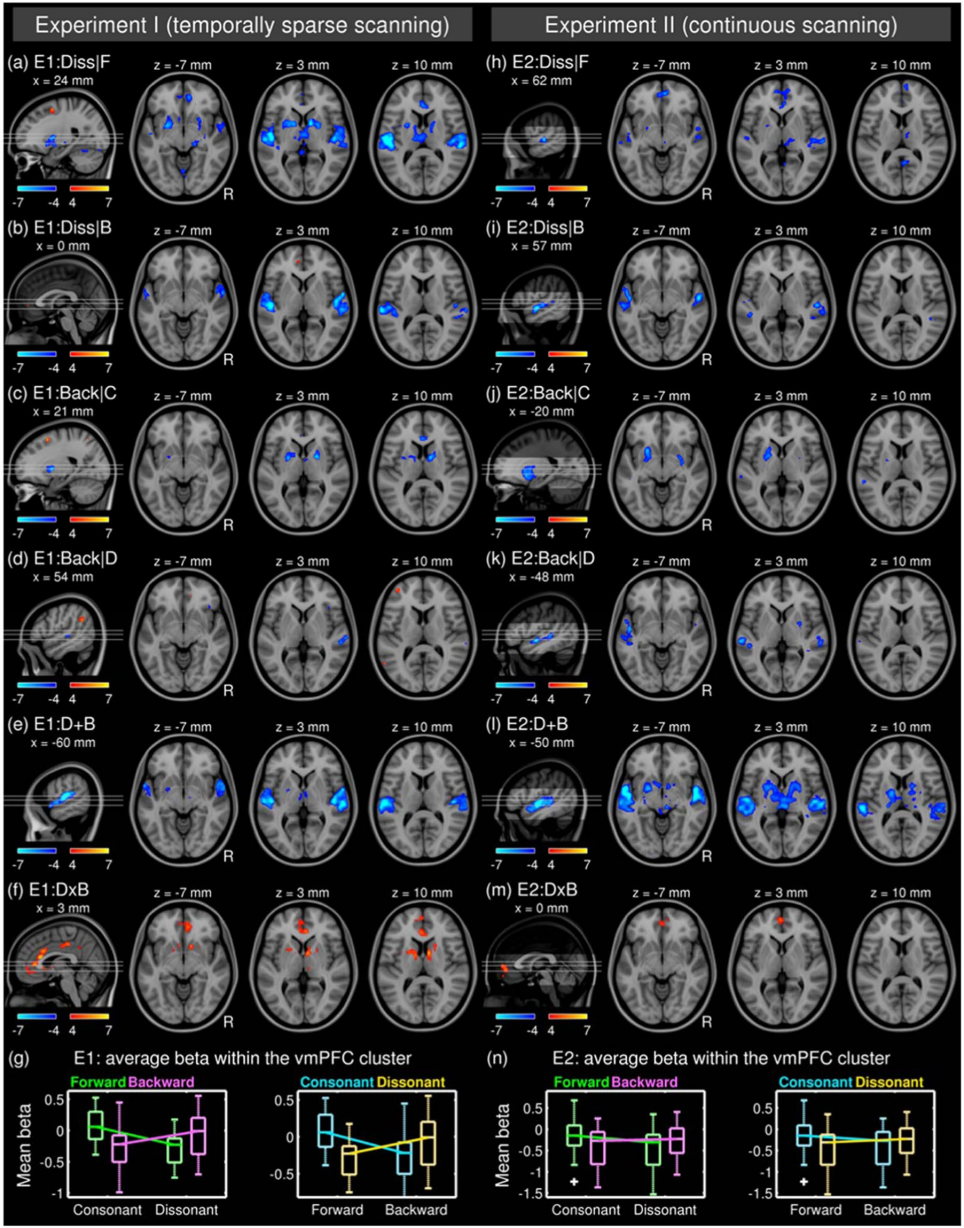
Comparison of the effects of disruption in musical structures in Experiment I (left column; degrees of freedom = 15) and Experiment II (right column; degrees of freedom = 22).

We analysed the Experiment II data set using the same processing pipeline except for the temporal processing (i.e. slice-timing correction and head motion covariates). The results from Experiment II were in good agreement with Experiment I, as shown in Fig. 5 and Table 3 (see Fig. S2 for all slices). Specifically, (1) deactivation in the bilateral STGs, vmPFC, and GPs due to dissonance alone (Fig. 5a,h), (2) selective deactivation only in the limbic area but not in the STGs due to reversal alone (Fig. 5c,j), (3) deactivation in the STGs due to joint disruption (Fig. 5e,j), and the positive interaction in the vmPFC (Fig. 5f,m). There were also noticeable differences between the experiments. However, significant differences between the experiments were mainly found in the auditory cortices (i.e., HG and PT), presumably related to the acoustic noise from gradient coils during continuous scanning, but not the left NAc in the interaction (see Fig. S3 for all slices).

**Table 3:** Significant clusters from Experiment II.

Structure name	Contrast value	Max *T* (22)	Max *Z*	Cluster *p*-value	Volume [cm^3^]	MNI-coord. [mm]		
						X	Y	Z
**(a) Partial effect of dissonance when forward**								
Right Superior Temporal Gyrus (posterior division)	−0.246	7.421	5.199	<10^−5^	5.1	62	−22	0
Right Frontal Medial Cortex	−0.359	6.400	4.760	<10^−5^	6.8	4	50	−8
Right Precuneus Cortex	−0.429	5.914	4.528	<10^−5^	1.5	4	−54	8
Right Subcallosal Cortex	−0.202	5.567	4.352	0.001	1.1	2	6	−15
Left Planum Polare	−0.315	5.427	4.278	<10^−5^	2.4	−48	−10	−2
Right Thalamus	−0.141	5.037	4.064	<10^−5^	2.6	7	−12	0
**(b) Partial effect of dissonance when backward**								
Right Superior Temporal Gyrus	−0.378	7.873	5.374	<10^−5^	6.7	57	−10	−5
Left Planum Polare	−0.371	6.329	4.727	<10^−5^	6.3	−48	−10	−5
**(c) Partial effect of reversal when consonant**								
Left Putamen	−0.153	4.762	3.906	0.004	0.9	−23	−2	−8
Left Putamen	−0.290	6.852	4.962	<10^−5^	6.2	−20	10	−10
Right Amygdala	−0.264	6.162	4.649	<10^−5^	2.8	27	0	−12
Subcallosal cortex	−0.142	5.974	4.557	0.048	0.7	10	−14	−15
Left Superior Temporal Gyrus (posterior division)	−0.222	5.187	4.148	0.004	1.2	−58	−37	10
**(d) Partial effect of reversal when dissonant**								
Left Planum Polare	−0.352	6.942	5.001	<10^−5^	6.0	−48	−7	−10
Right Temporal Pole	−0.250	5.700	4.420	0.002	1.3	54	6	−18
Right Superior Temporal Gyrus	−0.274	5.477	4.304	<10^−5^	2.2	60	−30	2
Right Putamen	−0.182	5.346	4.235	0.014	0.9	27	0	0
Left Inferior Frontal Gyrus	−0.238	4.099	3.495	0.031	0.8	−40	18	15
**(e) Joint effect of dissonance and reversal**								
Left Superior Temporal Gyrus (anterior division)	−0.552	9.785	6.016	<10^−5^	19.4	−50	−10	−5
Right Planum Polare	−0.597	9.436	5.909	<10^−5^	17.3	50	−2	−10
Right Frontal Orbital Cortex	−0.272	7.118	5.075	<10^−5^	1.7	32	28	−20
Brain-Stem	−0.253	7.076	5.057	<10^−5^	42.4	2	−27	−12
Left Cerebellum VI	−0.310	5.948	4.545	<10^−5^	1.8	−26	−60	−30
Left Occipital Pole	−0.384	5.223	4.168	0.009	0.9	−16	−100	−10
Right Cerebellum Crus I	−0.300	5.020	4.055	0.028	0.7	32	−82	−35
**(f) Interaction between dissonance and reversal**								
Left Paracingulate Gyrus	0.312	5.508	4.321	<10^−5^	3.2	0	48	−5
**(g) PPI between vmPFC and joint disruption**								
Right Frontal Pole	−0.183	5.545	4.340	0.016	0.7	30	53	2
Right Inferior Frontal Gyrus (pars triangularis)	−0.229	4.461	3.725	0.022	0.6	52	28	5

Anatomical nomenclatures are based on the Harvard-Oxford atlases provided in FSL (https://fsl.fmrib.ox.ac.uk/).

#### Psychophysiological interaction

In the vmPFC, the effect size (i.e. beta coefficient) of the FC condition was not significantly different from that of the BD condition in the Experiment I (*T*(15) = 1.01; *p* > 0.10) and Experiment II (*T*(22) = 1.22, *p* = 0.24). As mentioned earlier, this may look incongruent because two conditions were widely different in terms of acoustics and subjective rating. It may look more puzzling given that the vmPFC showed a strong decrease in BOLD activation due to the disruption of either a spectral or temporal structure. However, it is known that the vmPFC is engaged in widely various cognitive sub-processes^37^. Recent studies demonstrated reconfiguration of functional networks of the vmPFC due to external inputs^38,39^ and a level of arousal^40^. Given that, we suspected that the vmPFC might work similarly in terms of the activation level during the conditions of FC and BD but as a part of different functional networks. To test this idea, we analysed PPI between the vmPFC time series and the contrast between the FC and BD on the Experiment II data, which was acquired continuously.

**Figure 6:**
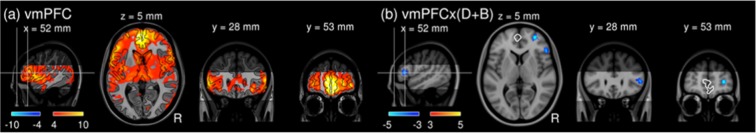
Functional connectivity of the vmPFC averaged across all conditions (**a**) and its modulation by the contrast between the “forward-consonant” (FC) and “backward-dissonant” (BD) conditions. (**b**) The seed cluster in the vmPFC is marked in white.

The results of the PPI analysis are shown in Fig. 6. The physical factor (i.e. correlation with the vmPFC time series) was found in extensive areas of the frontal cortices, temporal cortices, and subcortical structures (Fig. 6a). This functional connectivity of the vmPFC was reduced by the BD condition compared to the FC condition in the right IFG and FP (Fig. 6b; see Table 3g for statistics), supporting our conjecture on the vmPFC.

### Behavioural measures

**Figure 7:**
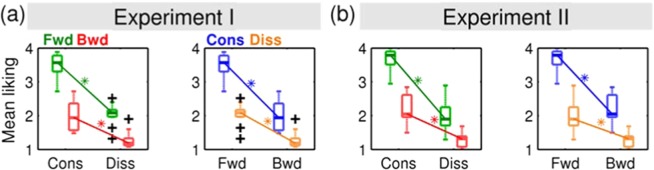
Effects of disruption of musical structures in subjective ratings of pleasantness. Abbreviation: Fwd, forward; bwd, backward; cons, consonant; diss, dissonant.

In correspondence to the observed changes in BOLD signal, we also found significant effects of disruptions and their interactions in the pleasantness ratings during scanning, as shown in Fig. 7 and Table 4. Notably, most participants rated the partially disrupted conditions (i.e. FD and BC) as “unpleasant” (mean rating of 2) without any particular preference for either (min *p* = 0.270). The interaction was also positive, but unlike the BOLD signal, the direction of effect was not changed so that the jointly disrupted condition (i.e. BD) was not more preferred than the partially disrupted conditions (i.e. FD or BC).

**Table 4:** Statistics of subjective ratings of pleasantness.

(a) Pleasantness rating (1 = very unpleasant, 4 = very pleasant)	Experiment I		Experiment II	
	Mean	Std.	Mean	Std.
Forward-Consonant	3.483	0.363	3.687	0.291
Forward-Dissonant	2.051	0.276	2.052	0.442
Backward-Consonant	2.002	0.443	2.180	0.422
Backward-Dissonant	1.278	0.221	1.280	0.197
**(b) Paired one-sample** ***t*-test**	**Effect size**	**p-value**	**Effect size**	**p-value**
Partial effect of dissonance when forward	−1.433	<10^−10^	−1.635	<10^−13^
Partial effect of dissonance when backward	−0.723	<10^−6^	−0.900	<10^−10^
Reversal effect of dissonance when consonant	−1.481	<10^−7^	−1.507	<10^−12^
Reversal effect of dissonance when dissonant	−0.772	<10^−6^	−0.773	<10^−7^
Joint effect of dissonance and reversal	−2.205	<10^−11^	−2.408	<10^−19^
Interaction between dissonance and reversal	0.709	<10^−5^	0.735	<10^−6^
Forward-Dissonance vs. Backward-Consonant	0.049	0.725	−0.128	0.270

Abbreviation: Std., standard deviation.

Since it was confirmed that the interaction between spectral and temporal structures is significant in terms of both BOLD signal and behavioural response, we further explored if there is a correlation between individual differences in neural and behavioural effects, as recently demonstrated in our previous study^10^. In other words, we looked at if an individual showed a small (or large) effect of the interaction in the pleasantness ratings also exhibited a small (or large) effect of the interaction in the BOLD signal. We tested the inter-subject correlation between contrast coefficients of the BOLD signal and the behavioural response for the interaction, but no significant correlation was found in either experiment (min *p* = 0.676).

## Discussion

In the current study, we found decreased BOLD signal in auditory and limbic systems (i.e. the bilateral STGs, vmPFC, NAc, Ptm, GP, amygdala, and thalami) due to partial disruption of both spectral and temporal organisation, which corresponded to decreased subjective ratings of pleasantness. These anatomical structures have been reported to be involved in the processing of music-induced emotions in the previous studies^3,8,9,11,20,41–45^. As we hypothesised, we found a significant, positive interaction between the disruptions in the temporal and spectral organisation of the music, which was localised in the fronto-limbic system (i.e. vmPFC, Nac, CdN, Ptm). Furthermore, we found a significant modulation of functional connectivity of the vmPFC by the combined disruption of the temporal and spectral organisation. Major findings were consistently observed in both fMRI data sets. In the following sections, we will discuss the relevance of our findings to neural mechanisms that contribute to the aesthetic appreciation of music.

### Partial effect of alteration of spectral and temporal structures

Dissonance is very often associated with unpleasant emotions not only by those who are familiar with Western music but also by infants^46,47^, people from an autochthonous African ethnic group with no prior exposure to polyphonic Western music^21^, in a documented case a non-human primate (i.e. a chimpanzee)^48^, and even chicken^49^. This suggests that an association between harmony and emotional valence is to some degree universal and innate, presumably related to the physical properties of tonal sounds and the network characteristics of the low-level auditory stream; for instance, encoding of beating and sensory consonance/dissonance by the neurons in the inferior colliculus^50^. An fMRI study^51^ revealed that certain acoustic information of aversive sound goes from the auditory cortex to the amygdala instead of directly from thalamic inputs, supporting the notion that certain complex aversive sounds need to be analysed at cortex level to induce negative emotional responses. A similar pathway was implicated from an intracranial recording of an epileptic patient, showing a cascade of information of dissonant harmony from the auditory cortex to the orbitofrontal cortex, ACC, and amygdala^52^ supporting that certain aversive acoustic information reaches the amygdala via the auditory cortex, presumably followed by feedback from the amygdala to the auditory cortex. We believe our finding of decreased BOLD activation in the auditory cortex and the limbic system also reflects such a communication between the auditory cortex and the limbic system that is related to “core liking”.

Constant alteration of a temporal structure in music seems to decrease activation in the limbic areas, such as the ventral striatum, hypothalamus, and the orbitofrontal cortex^17^, unlike a focal alteration that evokes a prediction error response in the IFG^20^. We also found reliable decreases in the bilateral putamina, which is known to be sensitive to emotional and motivational information^53^ and vastly studied in the context of decision-making^54^. It was theorised that the dorsal striatum (including the Ptm) contributes to an action selection in the context of decision-making, whereas the ventral striatum encodes reward value and prediction error^55^. More relevantly, a similar distinction was reported in the context of musical pleasure: The dorsal striatum encoded anticipation of musical pleasure, whereas the ventral striatum encoded experience of pleasure^43^. Therefore, the current finding of the decreased BOLD signal in the Ptm in response to the reversed excerpts seems to be related to impaired reward anticipation processes.

### Interaction between spectral and temporal structures of music

We found an interaction between spectral and temporal domains in areas including the ventral and dorsal striata, vmPFC, and ACC (prominently in Experiment I) that have been well associated with the reward processing^6^ and emotional appraisal^56–58^. In particular, we demonstrated that the direction of the effect of dissonance (or reversal) can be switched by the presence of reversal (or dissonance) in beta coefficients. In other words, it was shown that physically identical manipulations (e.g. dissonance) can produce opposite effects depending on the context (e.g. forward or backward) in those regions.

However, such an interaction (i.e. changes of the directions of effects) does not indicate that the disruption of the harmonic structure could be perceived as more pleasant when the temporal rules are already disrupted. In fact, the behavioural ratings showed that the two types of effects both contributed to rendering the musical excerpts more unpleasant. That is, a certain manipulation of a musical structure (e.g. dissonance) was similarly still unfavourable when the other musical structure (e.g. a sequential rule) was disrupted whereas it increased the BOLD signal in the vmPFC whereas it decreased the BOLD signal in the same region when it was presented with the other musical structure was intact.

One possible explanation of this might be a specific functionality of the vmPFC at integrating positive and negative emotions^59^, which was also subject to a computational imaging study^60^. Both human lesion and imaging studies point towards such an emotional functionality of the vmPFC. For example, patients with lesions in the vmPFC showed impairment in processing negative emotions^61,62^. In the current study, musical excerpts in their original forms (i.e. FC) and the most disrupted forms (i.e. BD) were rated as either very pleasant or very unpleasant while partially disrupted forms (i.e. FD and BC) were rated as (mildly) unpleasant. That is, relatively more (or less) intensive emotional valance might have been related to decreased (or increased) BOLD signal in the vmPFC. In fact, in a neuroimaging study^63^, where various types of musical emotions (e.g. peacefulness, joy, sadness and so on) were used, the vmPFC showed higher BOLD when a certain group of emotions (both positive and negative valance) was intensified, which also supports an integrative role of the vmPFC.

### Modulated functional connectivity of the fronto-limbic network

Another important finding in relation to the vmPFC was its differential functional connectivity with the IFG/FP when both musical structures were intact or disrupted although the BOLD activation levels were similar. We interpret this finding such that it suggests the vmPFC interacts with emotional processing (presumably in limbic areas) and cognitive processing (presumably in the IFG/FP) that is related to musical structures. The following findings support this notion:

1. The vmPFC has been mostly found to be involved in higher-order cognitive processing of emotional information. For instance, the vmPFC was found to be necessary in reward evaluation in decision-making^64,65^, nullifying learned conditioning^66,67^, emotional judgment of affective pictures^68^, and intensely pleasant emotions induced by music^4^. It has also been suggested that the vmPFC is involved in modulating autonomic processes^69^, which accompany emotional responses^70,71^.
2. The right IFG has been implicated in processing a series of chords^13^, melodies^72^, or even a periodic loop of random tones^73^, suggesting the IFG to be highly relevant for extracting regularity from sequential auditory inputs and forming expectation^74^.
3. Anatomically, in non-human primate models, direct connections between the frontal operculum and the basoventral PFC were found using a tracing technique^75^, suggesting a close relationship between the vmPFC and IFG.
4. The functional connectivity between the vmPFC and right IFG has been implicated in studies where emotional regulation is crucial. In a human study^64^, the functional connectivity between the vmPFC and IFG correlated with the performance level of a self-control task in relation to successfully inhibiting emotional responses. In another human study^76^, it was reported that an unstable interaction between the vmPFC and IFG was found in patients with anxiety disorder, which is suggestive of the IFG delivering higher-order sensory information to the vmPFC so that it can modulate the limbic system’s activities, that is, the vmPFC seems to work as a pivotal point that mediates between the limbic system and the frontal cortex in the regulation of emotion.

Taken together, it seems plausible that the vmPFC was engaged in cognitive processes that modulate emotional responses by differentially communicating with the right IFG and FP when listening to musical excerpts with varying musical structures.

### Technical limitation

It would be noteworthy that our manipulation in the temporal domain has some limitations. In this study, we were focused on sequential order in various temporal organisations of music, therefore reversal was one possible choice. In a study investigating phoneme encoding in EEG signals^77^, a similar manipulation (i.e., reversed speech) was used to disrupt the intelligibility of speech while preserving overall acoustic structures. However, in our experiments, reversing entire waveforms altered musical timbre together and did not alter beats and temporal intervals between notes while it remains unclear how these factors would interact with the sequential orders. Thus, more sophisticated temporal manipulations such as local reversal^78^ or quilting algorithm^79^ should be considered for more precise control of temporal structures for future studies.

## Conclusion

In the current study, we found a significant interaction between disruptions in the spectral and temporal structures of music in the brain activity of the fronto-limbic network. In particular, the vmPFC exhibited distinctive functional connectivity with the right IFG to altered spectral and temporal organisation of music, which is indicative of cognitive involvement in emotional processes, with the vmPFC as a pivotal node of a functional network mediating integration of cognition and emotion during music listening.

## Supplementary information

Supplementary information

## Data Availability

The datasets analysed in the current study are available upon reasonable request to the corresponding author.
