# Supplementary material for: Brain networks underlying aesthetic appreciation as modulated by interaction of the spectral and temporal organisations of music
Source: Sci Rep. 2019 Dec 19;9:19446. doi: 10.1038/s41598-019-55781-9 (PMC6923468; doi:10.1038/s41598-019-55781-9)
Supplement: Supplementary file 1 — Supplementary information [file 41598_2019_55781_MOESM1_ESM.pdf]

Supplementary Information

Brain networks underlying aesthetic appreciation as modulated by interaction of the spectral and temporal organisations of music

Seung-Goo Kim[1,2*], Karsten Mueller[1], Jöran Lepsien[1], Toralf Mildner[1], and Thomas Hans Fritz[1,3]

1 Max Planck Institute for Human Cognitive and Brain Sciences, Leipzig, Germany

2 Department of Psychology and Neuroscience, Duke University, Durham, NC, United States

3 Institute for Psychoacoustics and Electronic Music, University of Ghent, Ghent, Belgium

* Correspondence should be addressed to S.-G.K.

E-mail: sol@snu.ac.kr

Postal: 308 Research Drive, Durham, NC 27708, USA

Telephone: +1 919 660 0719

Supplementary figures legend

Figure S1. T-statistic maps (degrees of freedom = 15) on axial slices from the MNI152-coordinate z = -20 mm to 50 mm with a step of 2 mm for various contrasts in Experiment I ("E1"). The contrasts are (a) marginal effect of dissonance when played forward ("Diss|F"), (b) marginal effect of dissonance when played backward ("Diss|B"), (c) marginal effect of reversal when consonant ("Back|C"), (d) marginal effect of reversal when dissonant ("Back|D"), (e) difference between marginal effects ("B-D"; i.e., BC-FD), (f) joint effect of dissonance and reversal ("D+B"; i.e., BD-FC), (g) interaction between dissonance and reversal ("DxB"; i.e., FC-FD-BC+BD). Abbreviations: FC, forward-consonant; FD, forward-dissonant; BC, backward-consonant; BD, backward-dissonant.

Figure S2. T-statistic maps (degrees of freedom = 22) on axial slices from the MNI152-coordinate z = -20 mm to 20 mm with a step of 1.5 mm for various contrasts in Experiment II ("E2"). Note that the coverage of the EPI scans only covered the ventral half of the brain. The contrasts are identical to Figure S1.

Figure S3. T-statistic maps (degrees of freedom = 38) that compares effects between two experiments (E1-E2) on axial slices from the MNI152-coordinate z = -20 mm to 20 mm with a step of 3 mm. The comparisons were done in all contrasts shown in Figure S1.

Supplementary file legend

File S1. An MP3 audio file of four versions of a representative musical excerpt from J. S. Bach's Piano concerto No. 5 played by Glenn Gould (3 s for each condition).

Supplementary table

**Musical Excerpt Title**	**Composer**
Entree Courante	Anonymous
Piano concerto No. 5 in F-minor	Johann Sebastian Bach
Badinerie - Piano concerto No. 2 in Bb-minor	Johann Sebastian Bach
I got rhythm	Benny Goodman
Bourree (Overture No. 1, BWV 1066)	Johann Sebastian Bach
La Punalada	Francisco Canaro
Volte	Pierre Francisque Caroubel
Slavonic Dance No. 8 (Op. 46)	Antonin Dvorak
Happy Jigs	Flook
Blue in You	Friend N Fellow
Drummin Man	Gene Urupa
Jeepers Creepers	Gene Urupa
Zorba the Greek	Herb Alpert & The Tijuana Brass
Going Home Theme	Mark Knopfler

Soul Intro "The Chicken"	Jaco Pastorius
Rejouissance	Johann Sebastian Bach
Riulruairc	Leaba
Amarru	Santiago J Roux
Bucovovina	Shantel
Kicking Around	The Ventures

Table S1. Titles and composers of twenty musical excerpts used in the current study.
